# The Application of Next Generation Sequencing to the Understanding of Antibody Repertoires

**DOI:** 10.3389/fimmu.2013.00265

**Published:** 2013-09-11

**Authors:** Pascale Mathonet, Christopher G. Ullman

**Affiliations:** ^1^Isogenica Ltd., Chesterford Research Park, Essex, UK

**Keywords:** next generation sequencing, antibody, repertoire, antibodyome, humoral immune response, synthetic library, deep sequencing

## Abstract

In the decade since the human genome sequence was declared complete, the development of next generation sequencing (NGS) or “deep” sequencing to deliver cost-effective genomic sequencing has influenced advances beyond its primary application and changed the research landscape in many other areas. This review will survey recent applications of NGS which have broadened the understanding of natural antibody repertoires (the “antibodyome”) and how these evolve in response to viral infection. We will also report examples where deep sequencing of binding populations, derived from both natural and synthetic repertoires, have been used to benefit antibody engineering. This knowledge will ultimately lead to the design of more effective biological drugs and vaccines.

## Introduction

Since the human genome was declared complete in April 2003, the costs of genomic sequencing have been reduced by orders of magnitude and available tools exist for data analysis, so that access to massively parallel sequencing is no longer the exclusive realm of large genome centers. Instead, next generation sequencing (NGS) technologies such as 454 pyrosequencing and Illumina Solexa clonal bridge amplification methods have become widespread and can be operated by individual investigators, allowing the scope of their use to be expanded (1–7). In this review, we will focus attention toward how NGS can reveal the sequence space occupied by antibodies in their recognition of antigens. Specifically, we will concentrate on how NGS informs our knowledge of natural antibody repertoires and how the humoral immune system adapts to infection, how it can be used to improve the efficiency of screening and how it can be applied to the design of new systems for the discovery of novel biologics.

## The Development of Natural Antibody Repertoires

The sequencing of natural antibody or immunoglobulin (Ig) repertoires poses a different challenge to genomic sequencing, as the antibodyome (“the knowledge of the complete set of antibodies in an individual”) is in constant flux whilst the genome is relatively invariant. The diversity of the antibodyome in adult humans is estimated from the numbers of antibody expressing B cells within the body which is approximately 10^10^–10^11^. However, this population changes quickly with time and tissue distribution, resulting from daily turnover and replenishment of B cells, which potentially introduces new sequences (8). This sequence multiplicity is due to several natural mechanisms, the most important being somatic recombination and rearrangement of two or three sets of gene segments into a single unique gene. Each antibody is made of the products of two genes, encoding heavy and light chains, which provide further intricacy. In the case of the heavy chain gene, at least 56 variable (V), 23 diversity (D), and 6 joining (J) segments exist that are rearranged by RAG1 (recombination activating gene 1) and RAG2 recombinases in a process known as V(D)J recombination that brings VDJ segments together (light chain genes lack D regions). Imprecise fusion at the junctions from insertions and deletions through the activity of terminal deoxyribonucleotidyl transferase creates additional combinatorial diversity (junctional diversity) and the sequence from the V-D, through the D segment to the D-J junction (or V-J junction in light chains) is known as the complementarity determining region (CDR) 3 that is critical for the antibody’s antigen specificity (9, 10).

Therefore, most of the effort to understand human antibody repertoire has focused on deep sequencing of the CDR3 regions, especially the heavy chain CDR-H3, to understand repertoire diversity. The uniqueness of individual’s antibodyome was highlighted by results from sequencing these regions from two human subjects, which discerned a slight bias in the pairing of D and J but not in V-DJ. Also, between two individuals, there were CDR-H3s in common although this was a small fraction of the CDR-H3 diversity, estimated to be between 3 and 9 × 10^9^ (10). Sequencing efforts have demonstrated that certain V_H_ segments are over-represented in the natural human repertoire but it was uncertain whether this was determined by genetic, disease, age-related, or environmental factors (11, 12). However, a study on two monozygotic twin pairs demonstrated that whilst the variation in the naïve V_H_ and D_H_ segment use is strongly dictated by the individual’s germline genetic background, even in the case where one twin was affected by multiple sclerosis (MS), the CDR-H3 repertoires were highly specific to the individual. The authors suggested that even with common gene segment profiles, there is likely to be a different antibody response to common environmental exposure (13).

Interestingly, in contrast to the CDR-H3 repertoire, recent investigation in the rearrangement in the light chain CDR-L3 found that a surprisingly large proportion of CDR-L3 (more than 20%) was shared across individuals, which indicates that an intrinsic genetic mechanism is responsible for maintaining those sequences during human evolution (14).

Although the diversity of antibody repertoire is largely due to V(D)J rearrangement, the immune response is mediated by switching between Ig isotypes from IgM/IgD to the more specific and effective isotypes IgA and IgG, important for efficient response to infectious agents and induction of immune protection following vaccination. The results of a study that monitored Ig-isotypes from 14 healthy donors from different age groups and gender using NGS showed that the V(D)J recombination pattern did not seem to be affected by age, but there was a difference of isotype representation between young adults and elderly donors who displayed a reduction in class switch recombination (CSR) ability (15). This is in agreement with reduced vaccination efficacy observed in the elderly and was supported by an experiment in which the antibody repertoire was sequenced before and after influenza vaccination (16). It was found that older donors have fewer lineages; however, their antibodies prior to vaccination had a higher level of mutation. In a separate study of antibody classes and sequences derived from the peripheral blood cells (PBCs) from six young and six old donors, before and after influenza and pneumococcal vaccination, no age-related differences were found for IgG except for a higher mutation rate in the elderly and greater use of IgG2 (17). However, the most striking difference was in the IgA response, mostly IgA1 (associated with serum response), that showed a reduced, slower, clonal expansion. Both IgM and IgA had less hypermutations, but larger CDR-H3 regions, which implies that there is a defect in the mechanism of hypermutation in IgM and IgA that might affect antigen recognition in the old.

Species with fewer B cells have been used as model systems in an attempt to understand repertoire development. Zebrafish, which has approximately 3 × 10^5^ antibody producing B cells, some 10^5^-fold less than humans, were found to utilize between 50 and 86% of the 975 possible V(D)J combinations (18). Interestingly, individual fish shared a small number of identical heavy chain sequences. In a later article, it was shown that the early V(D)J repertoire between young fish was correlated due to antigen naivety but developed greater diversification on maturity through somatic hypermutation and antigen selection. The older fish had a higher junctional diversity due to insertion and deletions from greater expression of terminal deoxyribonucleotidyl transferase and the repertoire shifted from one in which mutated sequences are marginally expressed to one where these were highly expressed (19). As intimated from the later vaccination study of Jiang et al. above, it is tempting to speculate that similar events occur within humans as the immune system matures.

## Insights into the Immune Response Using NGS

Understanding how the human humoral immune system responds to infection is important for counteracting immune evasion and to enlighten the development of effective vaccines and antibody-based therapeutics. Recently, NGS has been used in this regard to help understand the natural maturation of broadly neutralizing antibodies (bnAbs) in HIV-1 infected individuals.

HIV-1 has evolved multiple mechanisms to evade the humoral response and 10–25% of infected individuals develop cross-reactive antibodies to a range of epitopes after several years of infection. Changes in one such example of HIV-1 bnAbs,VRC01-like antibodies, that recognize the CD4-binding site of HIV-1 gp120 have been followed by a combination of NGS and crystallography (20). The mature VRC01 antibody is remarkable as it develops over 70 mutations during the maturation process and can neutralize approximately 90% of HIV-1 isolates. By studying VRC01-like antibodies from patients, the authors observed a maturation process that converged structures for optimizing hydrophobic interactions that were precisely focused on an epitope for the initial site of gp120-CD4 interaction. The convergence in epitope recognition was accompanied by a divergence in antibody sequence identity, including the occurrence of chemically conservative changes in the paratopes and heavy chain revision or other mechanisms of B cell diversification, as suggested by sequencing of maturation intermediates (20). The heavy chains of the bnAbs had also the potential for promiscuity in their light chain pairing, but NGS could not elucidate the natural heavy and light chain pairings (discussed below). In a separate study, variants of bnAb 10E8, which recognizes a helix-turn-helix motif in the membrane-proximal external region of HIV-1 gp41, were identified with somatic mutation levels as high as 28%. Phylogenetic trees created from NGS and grid sampling were used to infer natural functional heavy and light chain pairings, but mismatched pairings led to greater autoreactivity (21). It is hypothesized that the high degree of somatic mutations in the HIV-1 bnAbs is seen as a consequence of an evasion strategy of HIV-1 where epitopes are poorly recognized by germline antibodies and thus studies have been conducted to identify appropriate intermediate and germline lineages through NGS, phage screening, and phylogenetic analysis with the hope of identifying better frameworks for antiviral bnAbs (22, 23).

In a similar investigation, peripheral blood mononuclear cells (PBMCs) were isolated from a healthy individual following the 2009 H1N1 influenza pandemic (24). Five antibodies were isolated as hybridomas that bound the Sa antigenic site of the globular head of hemagglutinin and had a broader spectrum against H1N1 strains than the 2D1 neutralizing antibody cloned from a survivor of the 1918 H1N1 pandemic. The antibodies shared the same heavy chain frameworks, V_H_3–7/J_H_6, but were derived from four independent clones that showed convergence in CDR-H3 properties and conservation of some somatic mutations, demonstrating an oligoclonal response against a viral antigen similar to those observed against HIV-1. Using 454 pyrosequencing, clones could still be identified, albeit rarely, in the peripheral blood of the donor 6 months after the initial blood draw for hybridoma generation. However, the V_H_3-7/J_H_6 segments represented large circulating phylogenies containing divergent unmutated germline sequences as well as mutated clones, supporting the view that these may assist in generating future immunological responses to the same or similar antigens (24). Interestingly, a non-NGS study of antibody responses to H1N1 infection used cell sorting, single cell RT-PCR, and monoclonal antibody expression to identify bnAbs that recognized the stalk or head domain of hemagglutinin. These antibodies also contained a high degree of somatic mutations per sequence (>19 on average) and a restricted V_H_ usage suggesting maturation and adaption to these epitopes on different influenza strains (25).

A study of New Zealand white rabbits immunized with hemocyanin, similarly showed an oligoclonal serum response with 34 antibodies grouping into 30 clonotypes likely to have been derived from different progenitor B cells. The repertoire was dominated by two to three V_H_ and two J_H_ families with a subpopulation containing a large number of mutations. The V_H_ response was consistent with the abundances observed for the V_H_ repertoire highlighting the importance of the cellular repertoire in determining humoral immunity. Seven days post-immunization, 16 out of 34 of the identified CDR-H3s in the serum repertoire map exclusively to sequences found in the PBC database, and may be derived from recently activated plasma blasts in transit to the bone marrow, whereas the remainder were likely to be expressed from plasma cells that had migrated to the bone marrow. There was also evidence of oxidative modification from mass spectrometry but it was not known if this was an *in vivo* posttranslational modification contributing to additional diversity in the antibody libraries (26). In another analysis of B cell distribution, the repertoire was compared on both sides of the blood-brain barrier (BBB) in MS and patients suffering other neurological diseases. In some patients, common V_H_ sequences were identified on both sides of the BBB, but the data indicated that only a few B cells migrate through the BBB are retained in the central nervous system (CNS). In MS patients the IGHV4 segment predominates, suggesting the framework to be particularly suited against MS antigens (27).

## Overcoming the Limitations of NGS in Antibody Repertoire Analysis and Its Utility in Screening

As highlighted in the examples above, a deficiency of NGS is that it is not currently possible to sequence both of the two chains of the antibody in a single read. Therefore, when using the common methods, no information on the natural V_H_:V_L_ pairing, which is crucial to discern native antibodies, can be obtained beyond inference from frequency analysis of sequencing separately both variable domains. However, despite this limitation, 21/27 scFvs constructed from pairing together the most abundant V_H_ and V_L_ genes from immunized mice were expressed in *E. coli* and bound antigen with nanomolar affinity. Yet, pairing differently ranked heavy and light variable domains in a full antibody format yielded a subnanomolar IgG in HEK 293F cells (28). Indeed, the method of repertoire mining of V_H_ and V_L_ abundances through NGS of splenocytes, isolated from immunized mice, was compared with a phage panning approach of the same cDNA. While both methods provided antibodies with comparable affinities, clones identified by repertoire mining showed higher selectivity for the antigen. Antibodies selected by phage display were barely detected by NGS, and conversely, mining the V repertoire identified antigen-specific antibodies that were not selected by phage display (29). This study demonstrated the expression bias of traditional phage display methods and the complementarity of using both approaches to isolate both rare and abundant binding sequences, thus supporting results from an earlier study by Ravn et al. (30). Here, NGS data were used to retrieve scFvs that could bind to the target with high affinity without the need for primary screening. Indeed the methods enabled the retention of clones that could have been lost during screening in small-scale soluble expression formats. A similar method has been used for screening an antigen in a more complex environment, where the antigen was not purified but displayed on the bacterial surface. NGS analysis of a scFv library that bound to bacterial cells expressing the target, versus a control population, provided information necessary to synthesize scFv binders to IL-6 (31).

A proteomics approach that combines high resolution LC-MS/MS analysis of purified and digested fragments of serum antibodies referenced against databases derived from the NGS reads of the B cell repertoire has been developed to provide more precise information for V_H_:V_L_ pairing (32). However, this is a difficult problem to solve using proteomics because V_H_ and V_L_ abundances do not correlate, due to an excess of V_L_ secreted into the serum, and the fact that V_L_ sequences have lower complexity which results in V_L_ sequences sharing partial identity (26).

Recently, an elegant solution to this problem has been described, which isolated more than 5 × 10^4^ single B cells individually in the microwells of a high-density microplate (33). Poly-dT beads were added to the wells and, after cell-lysis, the mRNA was captured on the beads and emulsified for cDNA synthesis. The V_H_:V_L_ pairs were linked by PCR and sequenced by paired-end long reads using Illumina technology. This experiment was performed on repertoires post-immunization for antigen-specific plasmablasts against tetanus toxoid and for memory B cells after influenza vaccination. Some of the V_H_:V_L_ pairs identified were expressed in IgG format and they all demonstrated affinities in the subnanomolar-nanomolar range.

## Application of NGS to Future Antibody Engineering

Antibody display libraries derived from human PBMCs or hybridomas immortalized from B cell populations have been successfully used in recent decades to isolate binders against a wide range of targets, despite a lack of detailed knowledge of the repertoires (34, 35). With the advent of NGS, analysis of the natural naïve repertoires from which libraries have been constructed has become possible.

In an early paper, the diversity of a phage displayed combinatorial library generated from the IgM repertoire of 654 healthy human donors was precisely quantified by deep sequencing pre- and post-selection using long-read pyrosequencing (12). Variable domain PCR amplicon and rolling circle amplified shotgun methods allowed an efficient assessment of diversity, as well as the correct assignment of heavy and light chain pairing. A novel application of Hidden Markov Model (HMM) accurately identified CDR regions. The sequencing results revealed that all germline families were present and a high degree of somatic mutations in CDR1 and CDR2 provided additional complexity to a library that was estimated to be similar in diversity to the number of transformants (3.5 × 10^10^). The library was subjected to panning against 16 targets and pairing preferences were observed for heavy and light chains. This information was used to produce combinatorial libraries that mimic the natural repertoire both in length and sequence diversity. A synthetic Fab library was fabricated in which all six CDRs are diversified by synthetic enzymatic codon addition method allowing precise control of amino acid additions at each position in the CDRs, to recapitulate those found in nature. The library was subjected to panning against a diverse panel of receptors, growth factors, antigens, enzymes, and peptides. Binders were isolated for all antigens with nanomolar affinities measured for 6 out of 10 antigens (36).

Larman et al. (37) describe an interesting synthetic approach where CDR sequences were designed using a HMM model of “contact” and “non-contact” states for amino acid positions based upon known antibody-antigen complexes. These sequences were synthesized in a releasable format on a DNA microarray, assembled into a single framework scFv library, panned by ribosome display against poliovirus receptor-related 4 (PVRL4) and the binding output submitted to NGS analysis. Of the top 25 most abundant clones post-selection, four were found to specifically bind human mammary epithelial cell (HMEC)-expressed PVRL4 by FACS-staining analysis.

Further scrutiny of natural systems, the use of modern synthetic approaches, surveying the enrichment process, and examining the resulting targeted antibody repertoire will better inform the design of next generation synthetic libraries (36, 38–40). This will undoubtedly improve the performance of synthetic libraries, many of which have been poor in functionality due to degenerate designs that do not respect loop length diversity, amino acid, or structural preferences of natural systems.

## Conclusion

Major challenges still remain in the use of NGS for antibody research with respect to reliably identifying the heavy and light chain pairs, as well as the bioinformatic analysis of the output. However, in a relatively short time span, NGS has impacted heavily on our understanding of the mechanism of the humoral response to viral insult, antibody clonal selection, and the chemical and structural nature of the binding landscape of the variable domains. Yet, whilst it has enabled researchers to take small steps forward in the quest for deriving the ultimate binders from their systems, NGS methods also highlight the need to learn more from both natural and synthetic repertoires. Sequencing combined with proteomics techniques will provide better resolution of the natural systems, and modern library synthesis methodologies will allow greater use of rational and combinatorial approaches. These will permit scientists to create designer libraries that will specifically address certain classes of antigens or determine greater biophysical stability, manufacturability, longer shelf-life, and improved pharmacokinetic and pharmacodynamics properties than existing antibody therapeutics.

## Conflict of Interest Statement

The authors declare that the research was conducted in the absence of any commercial or financial relationships that could be construed as a potential conflict of interest.
